# A Comprehensive Review of the Vascular Consequences of Diabetes in the Lower Extremities: Current Approaches to Management and Evaluation of Clinical Outcomes

**DOI:** 10.7759/cureus.47525

**Published:** 2023-10-23

**Authors:** Akanksha Yachmaneni, Suhas Jajoo, Chandrashekhar Mahakalkar, Shivani Kshirsagar, Simran Dhole

**Affiliations:** 1 General Surgery, Jawaharlal Nehru Medical College, Datta Meghe Institute of Higher Education & Research, Wardha, IND; 2 Surgery, Jawaharlal Nehru Medical College, Datta Meghe Institute of Higher Education & Research, Wardha, IND

**Keywords:** comprehensive review, pathophysiology, clinical outcomes, management strategies, lower extremities, diabetes-related vascular complications

## Abstract

Diabetes mellitus is a global health concern characterized by chronic hyperglycemia, and its vascular consequences in the lower extremities pose significant challenges for individuals living with the condition. This comprehensive review delves into the multifaceted landscape of diabetes-related vascular complications in the lower limbs, with a primary focus on current strategies for management and the evaluation of clinical outcomes. This review achieves several critical objectives by synthesizing existing knowledge and research findings. It elucidates the intricate pathophysiological mechanisms underpinning these complications, shedding light on the cellular and molecular processes involved. Additionally, it outlines clinical assessment and diagnostic strategies used to identify and stratify risk, ranging from cutting-edge imaging techniques to clinical examinations. The review comprehensively examines current management strategies, encompassing lifestyle modifications, pharmacological interventions, surgical procedures, and wound care practices. Moreover, it assesses and analyzes clinical outcomes, including limb salvage rates, amputation rates, and overall quality of life for individuals undergoing treatment. In addressing the challenges faced in managing these complications, this review aims to contribute to improved patient care. It proposes future research directions to enhance the management and outcomes of diabetes-related vascular consequences in the lower extremities.

## Introduction and background

Diabetes mellitus is a prevalent and growing global health concern. It is a chronic metabolic disorder characterized by hyperglycemia resulting from defects in insulin secretion, insulin action, or both. According to the International Diabetes Federation (IDF), an estimated 463 million adults were living with diabetes in 2019, projected to rise to 700 million by 2045. This exponential increase in diabetes incidence has far-reaching implications for public health systems, economies, and, most importantly, the lives of individuals affected [1].

Diabetes burdens healthcare systems considerably, with associated healthcare costs escalating because of complications and comorbidities. Among the various complications of diabetes, vascular consequences in the lower extremities pose a substantial challenge. These complications encompass a spectrum of conditions, ranging from peripheral arterial disease (PAD) to diabetic foot ulcers, and they often culminate in severe outcomes such as gangrene and amputations [2].

The impact of vascular complications on the lower extremities cannot be overstated. They are a leading cause of morbidity and mortality among individuals with diabetes. The reasons for the gravity of these complications are multifaceted. Firstly, diabetes-related vascular issues in the lower limbs impair an individual's mobility and quality of life and can lead to complications, including non-healing ulcers, infections, and limb-threatening ischemia. Secondly, lower extremity vascular complications often necessitate costly medical interventions, hospitalizations, and extended rehabilitation periods [3].

Moreover, the emotional and psychological toll on patients experiencing these complications is profound. The threat of amputation, coupled with chronic pain and disability, can lead to depression and decreased overall well-being. Therefore, understanding the mechanisms, diagnosis, management, and clinical outcomes associated with vascular consequences in the lower extremities is paramount. It is essential for healthcare providers, policymakers, and the general public to comprehend this in order to address the escalating burden of diabetes-related vascular complications [4]. The primary objective of this comprehensive review is to thoroughly explore the vascular complications afflicting the lower extremities of individuals with diabetes. These complications represent a significant and often debilitating aspect of diabetes management, encompassing conditions such as PAD, diabetic foot ulcers, gangrene, and the potential need for limb amputations. This review aims to achieve several critical goals by synthesizing existing knowledge and research findings.

## Review

Overview of diabetes types and prevalence

Type 1 Diabetes

Type 1 diabetes, often called juvenile diabetes, typically manifests in childhood or early adulthood. It results from an autoimmune reaction in which the body's immune system mistakenly targets and destroys the insulin-producing beta cells in the pancreas. This leads to an absolute insulin deficiency, necessitating lifelong insulin replacement therapy. Type 1 diabetes accounts for approximately 5-10% of all diabetes cases. Although it cannot be prevented, its onset can be sudden and requires careful management [5].

Type 2 Diabetes

Type 2 diabetes is the most common form of diabetes, comprising most cases worldwide. It primarily affects adults but can also develop in children and adolescents, particularly in cases of early-onset type 2 diabetes. Type 2 diabetes represents approximately 98% of global diabetes diagnoses, although this proportion varies widely among countries. This type is characterized by insulin resistance, where the body's cells do not respond effectively to insulin, and relative insulin deficiency, where the pancreas does not produce enough insulin to compensate for the resistance. Multiple factors contribute to its development, including genetics, obesity, physical inactivity, and poor dietary habits. Type 2 diabetes is often managed with lifestyle modifications, oral medications, and, in some cases, insulin therapy [6].

Gestational Diabetes

Gestational diabetes occurs during pregnancy, affecting about 7% of pregnant women. It is characterized by elevated blood glucose levels that develop or are first recognized during pregnancy. While it usually resolves after childbirth, the mother and child are at an increased risk of developing type 2 diabetes later in life. Proper management during pregnancy is essential to reduce the risk of complications for both the mother and the baby [7].

Other Forms

There are rarer forms of diabetes, including monogenic diabetes, which results from specific gene mutations affecting insulin production or function. Secondary diabetes can also occur due to other medical conditions (e.g., pancreatic diseases) or certain medications (e.g., corticosteroids) [8].

Pathophysiology of vascular complications in diabetes

Microvascular Complications

Diabetic Nephropathy: The pathogenesis of diabetic nephropathy involves several interconnected factors. Prolonged exposure to high blood sugar levels triggers events, including activating various signaling pathways and releasing pro-inflammatory molecules. These processes lead to structural and functional changes in the kidney's small blood vessels (glomerular capillaries). Over time, these changes result in the thickening of the glomerular basement membrane and accumulating extracellular matrix proteins, causing a decline in kidney function. The progression of diabetic nephropathy can ultimately lead to end-stage renal disease [9].

Diabetic retinopathy: Diabetic retinopathy is characterized by damage to the retina's small blood vessels (capillaries). Globally, the prevalence of diabetic retinopathy among diabetic patients is estimated to be 27%, which leads to 0.4 million blindness in the world. Chronic hyperglycemia and associated metabolic changes disrupt the normal regulation of blood flow in the retina. This leads to microaneurysms, hemorrhages, and the formation of abnormal blood vessels, which can leak fluid into the retina, causing vision impairment and, in advanced stages, blindness. Inflammatory processes and oxidative stress further contribute to retinal damage [10].

Diabetic neuropathy: Diabetic neuropathy results from nerve damage caused by prolonged exposure to high blood sugar levels and related metabolic disturbances. Approximately 50% of patients with diabetes will eventually develop neuropathy. Hyperglycemia can lead to the accumulation of toxic byproducts and oxidative stress, damaging nerve fibers and impairing nerve signaling. Additionally, reduced blood flow to nerves due to microvascular changes plays a role in the development of neuropathy. Diabetic neuropathy can manifest as sensory loss, pain, and autonomic dysfunction [11].

Macrovascular Complications

Coronary artery disease (CAD): Individuals with diabetes have an increased risk of developing atherosclerosis, a condition characterized by plaque buildup (atherosclerotic lesions) within the coronary arteries. This process is accelerated by chronic hyperglycemia, insulin resistance, dyslipidemia (abnormal lipid profiles), and inflammation. Atherosclerotic plaques can obstruct blood flow to the heart, leading to angina (chest pain) or myocardial infarction (heart attack) [12].

Stroke: Diabetes is a major risk factor for ischemic strokes, which occur when a blood clot obstructs a cerebral artery, depriving a portion of the brain of oxygen and nutrients. Hyperglycemia contributes to the development of atherosclerosis in the cerebral arteries and promotes the formation of blood clots. Additionally, diabetes-induced microvascular damage in the brain's small vessels can impair cerebral circulation and increase the risk of stroke [13].

PAD: The pathophysiology of PAD in diabetes is closely linked to atherosclerosis in the peripheral arteries, particularly those supplying the lower extremities. Atherosclerotic plaques narrow and stiffen these arteries, reducing blood flow and oxygen delivery to the legs and feet. Diabetes-related factors, such as hyperglycemia and inflammation, accelerate the progression of atherosclerosis in peripheral arteries, leading to symptoms like intermittent claudication, non-healing ulcers, and an increased risk of limb amputation [14].

Lower Extremity Vascular Complications

PAD: As mentioned above, PAD involves the narrowing or blockage of arteries in areas outside of the heart and brain, commonly affecting the lower extremities in individuals with diabetes. This condition reduces blood flow to the legs and feet, leading to symptoms such as intermittent claudication (leg pain while walking), cold or pale extremities, and weakened pulses. Impaired blood flow can also result in non-healing wounds, particularly on the toes or feet. In severe cases, PAD can progress to critical limb ischemia (CLI), where there is an imminent risk of tissue death, necessitating urgent intervention to prevent amputation [15].

Diabetic foot ulcers: Diabetic foot ulcers are open sores that typically develop on the bottom of the foot or areas with bony prominences. They are primarily caused by a combination of factors, including poor circulation, neuropathy (nerve damage), and trauma. Foot ulcers are especially concerning because they can become infected, leading to cellulitis or abscess formation. These ulcers may progress to more severe complications like gangrene [16].

Gangrene: This is a severe and potentially life-threatening complication resulting from body tissue death due to a lack of blood flow and oxygen. In individuals with diabetes, gangrene often affects the lower extremities, where impaired circulation and neuropathy increase the risk. Two main types of gangrene are encountered: dry gangrene, characterized by tissue dryness, shriveling, and blackening, and wet gangrene, which involves tissue infection and liquefaction. Gangrene necessitates immediate medical attention, as it can lead to systemic infection and sepsis if left untreated [17].

Amputation: Lower extremity amputation represents the most extreme consequence of uncontrolled lower extremity vascular complications. When infection and tissue death progress unchecked, amputation may be the only option to save the individual's life. Amputations significantly impact a person's mobility, independence, and overall quality of life. They are also associated with increased mortality rates and heightened healthcare costs [18].

Clinical assessment and diagnosis

Screening and Early Detection of Lower Extremity Vascular Complications

Foot examinations: Regular foot examinations conducted by healthcare providers are a cornerstone of early detection. These exams are particularly essential for individuals at high risk due to diabetes. During a foot examination, healthcare professionals assess for several key signs, including skin changes (such as dryness, calluses, or ulcers), loss of sensation (neuropathy), and deformities (such as hammer toes or Charcot's joint). Identifying these signs early can lead to timely interventions to prevent further complications [19].

Vascular assessments: The ankle-brachial index (ABI) measurement is a non-invasive and highly informative test. It involves comparing the blood pressure in the ankle to that in the arm. A lower ABI indicates reduced blood flow to the lower extremities, indicative of PAD. Identifying PAD early through ABI testing allows for initiating appropriate treatments, lifestyle modifications, and risk factor management [20].

Neurological assessments: Peripheral neuropathy is a common complication of diabetes that involves the loss of sensation in the extremities. Healthcare providers often employ tools like the monofilament test, which assesses a patient's ability to feel a specific level of pressure on the foot. Detecting neuropathy early is crucial because it is a significant risk factor for developing foot ulcers. Early intervention can help prevent the progression of neuropathy and the subsequent development of ulcers [21].

Regular self-examinations: Besides professional assessments, individuals with diabetes are encouraged to actively participate in their care by inspecting their feet daily. Regular self-examinations help patients identify changes or concerns promptly. This empowers individuals to take immediate action, such as reporting any wounds or abnormalities to their healthcare provider. Self-examinations also promote awareness and adherence to foot care practices, crucial in preventing complications [22].

Risk Factor Assessment (Glycemic Control, Blood Pressure, Lipids)

The assessment and management of risk factors are pivotal in preventing and mitigating lower extremity vascular complications, which are often associated with conditions like diabetes and atherosclerosis. These risk factors encompass glycemic control, blood pressure management, and lipid control, each playing a significant role in the overall health of the vascular system and the prevention of complications [23].

Glycemic control is of paramount importance, particularly in individuals with diabetes. Elevated blood glucose levels can damage small and large blood vessels, increasing the risk of microvascular complications (such as diabetic neuropathy and nephropathy) and macrovascular complications (like PAD). Effective glycemic control involves maintaining blood glucose levels within a target range through medications, insulin therapy, and lifestyle modifications. Consistently managed blood sugar levels help to reduce the likelihood of vascular damage and promote overall vascular health [24].

Hypertension, or high blood pressure, is a common comorbidity in individuals with diabetes and is a significant risk factor for vascular complications. Elevated blood pressure can accelerate the progression of atherosclerosis, leading to arterial narrowing and increasing the risk of PAD. Managing blood pressure through lifestyle changes (such as a heart-healthy diet, regular exercise, and stress reduction) and medications when necessary is essential. Effective blood pressure control prevents cardiovascular complications and reduces the strain on blood vessels throughout the body, including those in the lower extremities [25].

Lipid control is another critical aspect of preventing lower extremity vascular complications. Elevated levels of cholesterol and triglycerides can contribute to the development of atherosclerosis, a leading cause of PAD. Statin medications, dietary modifications, and lifestyle changes manage lipid levels effectively. Lowering cholesterol and triglyceride levels can slow the progression of atherosclerosis, reduce the risk of vascular blockages, and ultimately help prevent complications related to poor blood flow to the lower extremities [26].

Role of Imaging Techniques (Angiography, Ultrasound, MRI) in Diagnosis

Imaging techniques are crucial in diagnosing and evaluating lower extremity vascular complications, offering valuable insights into the condition and guiding treatment decisions. Three primary imaging modalities, angiography, ultrasound (Doppler ultrasound), and MRI, are indispensable [27].

Angiography, including digital subtraction angiography (DSA), is a powerful diagnostic tool that provides highly detailed images of blood vessels. It is beneficial in assessing the extent and location of arterial blockages in conditions like PAD. A contrast dye is injected into the bloodstream during angiography, and X-ray images are captured to visualize the blood vessels. This procedure allows clinicians to precisely identify the location and severity of arterial obstructions, enabling them to plan interventions such as angioplasty or stent placement [28].

Doppler ultrasound, on the other hand, is a non-invasive imaging technique that assesses blood flow in both arteries and veins. It is precious for detecting stenosis (narrowing of blood vessels), occlusions (blockages), and abnormal blood flow patterns. Doppler ultrasound uses sound waves to create real-time images of blood flow, making it a safe and effective method for diagnosing vascular complications. This technique is commonly employed to assess blood flow in the lower extremities, aiding in the early detection of conditions like PAD and venous thrombosis [29].

MRI offers a radiation-free alternative to angiography for evaluating the vascular system. MRI provides comprehensive and detailed images of both soft tissue and vascular structures, making it particularly valuable in diagnosing vascular abnormalities in diabetic patients who may be at a higher risk for lower extremity complications. MRI can visualize blood vessels, identify areas of stenosis or blockages, and assess the overall condition of the vascular system. Additionally, MRI can be combined with contrast agents to enhance the visibility of blood vessels, further improving diagnostic accuracy [30].

Clinical Signs and Symptoms of Lower Extremity Vascular Complications

Recognizing clinical signs and symptoms is paramount in detecting and managing lower extremity vascular complications early. First and foremost, PAD presents with distinctive indicators. Patients often complain of intermittent claudication, a condition characterized by leg pain that arises during physical activity, mainly while walking, due to inadequate blood flow to the lower limbs. Additionally, PAD manifests as cold, pale, or bluish extremities, indicating poor circulation and weakened pulses in the lower limbs, reflecting reduced blood flow through the arteries. Moreover, non-healing wounds, particularly those on the toes or feet, can be a red flag for PAD, as insufficient blood supply impairs the body's natural healing processes [31].

Diabetic foot ulcers represent another critical concern. These ulcers are characterized by open sores that tend to develop on the bottom of the feet or near bony prominences. Detecting diabetic foot ulcers early can be challenging because they are often painless due to neuropathy, which is common in diabetes. This absence of pain can delay diagnosis, making regular foot examinations essential for individuals with diabetes. Additionally, signs such as redness, swelling, or discharge from the ulcer should raise immediate concerns and prompt medical evaluation [32].

Gangrene, a severe complication, presents in two primary forms: dry and wet gangrene. Dry gangrene is characterized by dry, shriveled, and black tissue, while wet gangrene involves swollen, moist, and discolored tissue, often accompanied by infection. Depending on the type and progression of gangrene, patients may experience severe pain or numbness in the affected areas. Both types necessitate urgent medical attention to prevent the spread of infection and further tissue damage [17].

In extreme cases where complications like gangrene or non-healing ulcers reach advanced stages and conventional treatments prove ineffective, amputation may be indicated. Amputation involves the surgical removal of the affected limb or part of the limb, aiming to save the patient's life or prevent the spread of infection to other parts of the body. Timely recognizing the clinical signs and symptoms associated with these lower extremity vascular complications is crucial for initiating prompt interventions, ultimately improving patient outcomes and quality of life. Regular check-ups, especially for individuals with risk factors like diabetes or a history of vascular disease, are essential for early detection and management [33].

Current management strategies

Lifestyle Modifications

Diet and nutrition: Lifestyle modifications are fundamental in diabetes management, with dietary changes being a cornerstone for achieving glycemic control and overall health. Carbohydrate management is a key strategy involving monitoring carbohydrate intake, opting for complex carbohydrates over simple sugars, and distributing meals evenly throughout the day. This approach helps to prevent rapid spikes in blood sugar levels. Encouraging healthy eating patterns is essential, emphasizing a balanced diet that includes an abundance of vegetables, whole grains, lean proteins, and healthy fats, all of which contribute to better blood sugar regulation and overall well-being. Portion control is another vital component, educating individuals with diabetes about appropriate portion sizes and the significance of moderation in their dietary choices. Monitoring blood sugar levels regularly is crucial, enabling individuals to make real-time adjustments to their diet and insulin doses as needed, thus maintaining optimal glycemic control [34].

Exercise and physical activity: Regular physical activity is a cornerstone of diabetes management, offering many benefits. Engaging in aerobic exercises, such as brisk walking, swimming, or cycling, can enhance insulin sensitivity, making it easier for cells to absorb and utilize glucose. Moreover, it promotes cardiovascular health, reducing the risk of heart disease, which is more prevalent in individuals with diabetes. Strength training, another essential aspect of physical activity, aids in glycemic control by increasing muscle mass and improving metabolism and glucose utilization. Additionally, flexibility and balance training are valuable exercises for individuals with diabetes, as they help prevent injuries and complications related to neuropathy and foot health. Regular physical activity supports blood sugar management and contributes to overall fitness and well-being, making it an indispensable component of a diabetes care plan. Tailoring exercise routines to an individual's fitness level and preferences ensures adherence and long-term success in managing diabetes through lifestyle modifications [35].

Pharmacological Interventions

Antidiabetic medications: Diabetes management often involves using various classes of medications to control blood sugar levels. Oral antidiabetic drugs constitute a fundamental component of this treatment regimen, encompassing metformin, sulfonylureas, dipeptidyl peptidase 4 (DPP-4) inhibitors, and sodium-glucose cotransporter-2 (SGLT2) inhibitors. These drugs, each with distinct mechanisms of action, are typically prescribed based on the patient's specific needs and response to treatment. Metformin, for instance, enhances insulin sensitivity and decreases glucose production in the liver. Sulfonylureas stimulate insulin secretion from the pancreas, while DPP-4 and SGLT-2 inhibitors modulate glucose metabolism differently. Additionally, insulin therapy may be necessary for individuals with type 1 diabetes and those with type 2 diabetes who cannot achieve target blood sugar levels with oral medications alone. Whether administered via injections or insulin pumps, insulin is vital for regulating blood glucose effectively [36].

Medications for vascular risk reduction: Diabetes is associated with an increased risk of cardiovascular complications, making it crucial to address vascular risk factors through pharmacological interventions. Physicians often prescribe statins, cholesterol-lowering medications that help manage lipid profiles and reduce the risk of atherosclerosis-related complications such as heart attacks and strokes. These drugs play a pivotal role in preventing the progression of cardiovascular disease in diabetic patients. In addition to statins, antiplatelet agents like aspirin are commonly utilized to reduce the risk of blood clot formation and subsequent cardiovascular events in individuals with diabetes who are at a higher risk of developing thrombotic complications. These medications collectively form an essential part of diabetes management by controlling blood sugar and safeguarding against the detrimental effects of diabetes on the cardiovascular system. Individualized treatment plans are established based on the patient's overall health, comorbidities, and risk factors, ensuring comprehensive care for those with diabetes [37].

Surgical and Interventional Treatments

Angioplasty: Angioplasty is a minimally invasive procedure to treat narrowed or blocked arteries in the lower extremities. During angioplasty, a catheter with a deflated balloon at its tip is inserted into the affected artery. Once the catheter reaches the site of the blockage, the balloon is inflated, compressing the plaque or blockage against the artery walls, widening the vessel, and restoring blood flow. This procedure helps alleviate symptoms such as leg pain and promotes improved circulation to the affected limb [38].

Stent placement: In some cases, stent placement may follow angioplasty. A stent is a mesh-like device made of metal or other materials inserted into the treated artery to help keep it open. Stents provide structural support to the artery and prevent it from collapsing or narrowing again. They are instrumental in cases where the artery is at risk of restenosis (re-narrowing) following angioplasty [39].

Bypass Surgery

Bypass surgery is a more extensive surgical procedure used when the blockages in the arteries are severe or when angioplasty and stenting are insufficient to restore adequate blood flow. During bypass surgery, a vascular surgeon creates a new pathway for blood to flow around an artery's blocked or narrowed segment. This is typically achieved by using a vein or synthetic graft (graft material) to bypass the affected portion of the artery. By rerouting blood flow, bypass surgery restores oxygen and nutrient delivery to the tissues downstream of the blockage, relieving symptoms and promoting tissue healing [40].

Wound Care and Management of Diabetic Foot Ulcers

Debridement: Debridement is a fundamental step in wound care, involving the removal of dead, damaged, or infected tissue from the ulcer site. This process helps create a clean wound bed, which is essential for healing. Debridement can be performed using various methods, including sharp debridement (surgical removal), enzymatic debridement (application of topical enzymes to break down dead tissue), or autolytic debridement (allowing the body's natural processes to break down tissue). Regular debridement promotes wound healing and reduces the risk of infection and further complications [41].

Infection control: Diabetic foot ulcers are susceptible to infection due to compromised blood flow and impaired immune responses. In cases where infection is present or suspected, antibiotics may be prescribed to treat or prevent the spread of infection. Effective infection control is essential to prevent more severe complications, such as cellulitis, abscess formation, or osteomyelitis (bone infection). Timely and targeted antibiotic therapy is crucial in managing infected diabetic foot ulcers [42].

Offloading: Offloading is a strategy used to relieve pressure on diabetic foot ulcers and reduce the risk of further damage. It involves redistributing weight away from the affected area to facilitate healing. Specialized footwear, custom orthotics, casts, or braces may be used to offload pressure and reduce friction on the ulcer. Offloading promotes wound closure and helps prevent the recurrence of ulcers in high-risk areas, such as the plantar surface of the foot. Proper offloading is key to diabetic foot ulcer management and prevention [43].

Advanced wound dressings: Advanced dressings promote wound healing by creating an optimal environment for tissue regeneration. These dressings may incorporate growth factors, antimicrobial agents, or other therapeutic substances that aid healing. For example, dressings containing growth factors can stimulate the formation of new blood vessels and tissue, accelerating wound closure. Advanced wound dressings are chosen based on the ulcer's characteristics and the patient's specific needs. They are crucial in optimizing wound healing and minimizing complications [44].

Patient Education and Self-Care

Foot care education: Foot care education is crucial in preventing diabetic foot ulcers and complications. Teaching patients how to inspect their feet daily helps them identify early signs of cuts, blisters, or sores, allowing prompt intervention. Proper hygiene practices, such as washing and drying feet carefully, are essential to prevent infections. Educating patients about selecting appropriate footwear, including well-fitting shoes and diabetic-friendly socks, helps minimize the risk of pressure sores and injuries. Overall, foot care education empowers patients to actively prevent foot-related complications and maintain their health [45].

Medication adherence: Ensuring patients understand their medications and their role in diabetes management is critical for medication adherence. Educating patients about their prescribed medications, including dosage, timing, and potential side effects, promotes compliance. Patients who comprehend the importance of adhering to their medication regimens are likelier to achieve and maintain target blood glucose levels, reducing the risk of vascular complications. Adequate medication adherence can significantly impact overall health outcomes [46].

Blood sugar monitoring: Education on blood sugar monitoring is essential for individuals with diabetes. Teaching patients to use glucometers and understand the significance of regular monitoring empowers them to manage their condition effectively. Monitoring blood glucose levels helps patients and healthcare providers track the impact of lifestyle choices, dietary habits, and medication adjustments on glycemic control. This information enables timely interventions to prevent hyperglycemia or hypoglycemia, reducing the risk of vascular complications associated with poorly controlled diabetes [47].

Lifestyle counseling: Lifestyle counseling encompasses guidance on adopting and maintaining healthy habits. Patients with diabetes-related vascular complications benefit from learning about nutrition, physical activity, stress management, and coping strategies. Lifestyle modifications, such as a balanced diet, regular exercise, and stress reduction techniques, can help improve blood sugar control and overall vascular health. Patient education on these topics equips individuals with the knowledge and tools to make informed decisions and foster healthier lifestyles [48].

Evaluation of clinical outcomes

Importance of Assessing Treatment Outcomes

Quality of care: Monitoring treatment outcomes is essential to providing high-quality healthcare. It allows healthcare providers to gauge the effectiveness of their interventions and ensure that patients receive the best possible care. By systematically evaluating outcomes, healthcare teams can identify areas for improvement in care delivery, refine treatment protocols, and adopt evidence-based practices. This commitment to continuous quality improvement ultimately enhances patient outcomes and healthcare quality [49].

Patient-centred care: Assessing treatment outcomes embraces the patient's perspective and fosters patient-centred care. It recognizes that healthcare decisions should be made collaboratively, considering everyone’s unique needs, preferences, and goals. When outcomes are considered from the patient's viewpoint, it becomes possible to tailor treatment plans to align with their preferences and values. This personalized approach promotes patient engagement, shared decision-making, and a more potent therapeutic alliance between patients and healthcare providers [50].

Prevention of complications: Tracking treatment outcomes is instrumental in identifying complications at an early stage. Whether monitoring blood glucose levels in diabetes management or assessing wound healing in vascular care, outcomes measurement enables healthcare providers to promptly detect signs of deterioration or recurrence. Early intervention is crucial for preventing the progression of vascular issues and reducing the risk of complications such as infections and amputations. By regularly assessing outcomes, healthcare teams can make timely adjustments to treatment plans and implement preventive measures to safeguard patients' well-being [51].

Resource allocation: In healthcare systems with limited resources, assessing treatment outcomes is vital for efficient resource allocation. It helps inform decisions about which treatments and interventions yield the best results and benefit patients most significantly. By identifying and prioritizing the most effective and cost-effective treatments, healthcare systems can allocate resources optimally, ensuring that patient care is practical and efficient. This data-driven resource allocation approach enhances healthcare delivery's sustainability and equity [52].

Clinical Endpoints and Measures

Limb salvage rates: Limb salvage rates are a critical measure that indicates the success of medical interventions and therapies in preventing limb amputations. This metric quantifies the proportion of patients who retain their limbs without needing amputation. The calculation involves dividing the number of patients who did not undergo amputation by the total number of patients in a specific population or cohort. Limb salvage rates are crucial because they directly reflect the ability of healthcare interventions to preserve patients' mobility, functionality, and overall quality of life. High limb salvage rates indicate successful treatment and prevention of severe complications [53].

Amputation rates: Amputation rates represent the percentage of patients undergoing limb amputations due to diabetes-related vascular complications. A high amputation rate suggests that interventions and treatments were ineffective in preventing the progression of vascular disease and its associated consequences. This measurement is obtained by dividing the number of patients who underwent amputation by the total number of patients in a specific population or cohort. Reducing amputation rates is a primary goal in managing diabetes-related vascular complications, as amputations significantly impact patients' independence and quality of life [54].

Quality of life: Quality of life assessment is a holistic measure that evaluates the overall well-being and life satisfaction of individuals undergoing treatment for lower extremity vascular complications. It considers various aspects of patients' lives, including physical, psychological, and social dimensions. Quality of life is typically assessed using validated scales and questionnaires, such as the Short Form Health Survey (SF-36), or disease-specific instruments like the Diabetic Foot Ulcer Scale (DFS). These tools provide a quantifiable way to measure and compare patients' quality of life before and after treatment. Improvements in quality-of-life metrics demonstrate the positive impact of interventions on patients' physical functioning, pain levels, emotional well-being, and social interactions [55].

Long-Term Follow-up and Monitoring

Continued surveillance: Regular check-ups and ongoing surveillance are essential to monitor patients for the recurrence of complications or the development of new issues. This includes routine assessments of vascular health, wound status, and overall foot health. These assessments help healthcare providers detect complications at their earliest stages, allowing for timely intervention and prevention of further deterioration. Patients with a history of vascular complications, such as diabetic foot ulcers or peripheral arterial disease, may benefit from a structured surveillance program to promptly address emerging problems [56].

Glycemic control: Long-term monitoring of blood glucose levels is critical to maintain target ranges and prevent further vascular complications. Patients with diabetes must work closely with healthcare providers to manage their blood sugar effectively. Continuous glucose monitoring (CGM) systems and regular A1c tests can provide valuable insights into glycemic control over time. Adjustments to medication regimens and lifestyle modifications may be necessary to achieve and maintain optimal blood sugar levels [57].

Medication management: Ensuring patients adhere to prescribed medications is an ongoing concern. Medication management includes monitoring adherence and assessing the need for adjustments over time. Medication regimens may change based on a patient's response, side effects, or evolving medical conditions. Regular medication reviews with healthcare providers help optimize treatment plans and minimize the risk of medication-related complications [58].

Rehabilitation: Patients who have undergone amputations or experienced severe vascular complications often require physical therapy and rehabilitation programs. These programs aim to enhance mobility, functional independence, and quality of life. Rehabilitation may include prosthetic training, gait training, strength and flexibility exercises, and wound care education. Long-term follow-up ensures that patients receive the ongoing support and interventions needed to maximize their recovery potential [59].

Patient education: Patient education is integral to long-term follow-up and monitoring. Reinforcing self-care practices and educating patients on recognizing early signs of complications are essential. Patients should be informed about the importance of daily foot inspections, proper wound care, and the significance of seeking prompt medical attention if they notice any concerning changes. Empowering patients with the knowledge and skills to manage their condition effectively can significantly reduce the risk of complications and improve their long-term outcomes [60].

Challenges and future directions

Barriers to Effective Management

Late diagnosis: Late diagnosis is a significant barrier to effective management. Many individuals with diabetes-related vascular complications, such as PAD or diabetic foot ulcers, are not diagnosed until the complications have advanced. This late diagnosis limits the available treatment options and often results in more severe outcomes, including the need for amputations. Timely diagnosis is essential for implementing interventions that can prevent or mitigate the progression of complications [61].

Health disparities: Health disparities play a significant role in limiting access to effective management. Marginalized populations, including racial and ethnic minorities, individuals with low socioeconomic status, and those living in underserved areas, often face healthcare services and resource barriers. These disparities can lead to delayed diagnosis, inadequate preventive care, and limited access to specialized treatments, exacerbating the burden of vascular complications in these communities [62].

Non-adherence: Patient non-compliance with treatment plans is a persistent challenge in managing diabetes-related vascular complications. This non-adherence can manifest as neglecting to take prescribed medications, failing to make necessary lifestyle changes (such as dietary modifications and exercise), or not following wound care protocols. Non-adherence can be driven by various factors, including forgetfulness, financial constraints, lack of understanding about the importance of treatment, or even psychological barriers. Non-compliance significantly hinders the effectiveness of management strategies and leads to suboptimal outcomes [63].

Lack of awareness: Low or no awareness about the seriousness of vascular complications is another barrier. Both patients and healthcare providers may underestimate the potential consequences until complications reach advanced stages. This underestimation can delay necessary interventions and preventive measures. Raising awareness about the risks, warning signs, and importance of early detection and management is crucial for addressing this barrier [64].

Resource constraints: In some regions, limited healthcare infrastructure, resources, and skilled professionals can hinder comprehensive care for diabetes-related vascular complications. This includes inadequate access to vascular specialists, wound care centers, and diagnostic equipment. Resource constraints can result in delayed diagnosis and suboptimal treatment options for affected individuals, particularly in rural or economically disadvantaged areas [65].

Emerging Therapies and Technologies

Advanced wound healing: Research in wound care is continually advancing with the development of innovative wound dressings, growth factors, and stem cell therapies. These approaches aim to accelerate wound healing, reduce the risk of infection, and ultimately lower the likelihood of amputation. Advanced wound dressings may incorporate materials like hydrogels, nanofibers, or biodegradable scaffolds to provide an optimal environment for tissue regeneration. Growth factors and stem cell therapies can promote tissue repair and angiogenesis, stimulating the formation of new blood vessels to support wound healing [66].

Peripheral arterial interventions: Advancements in minimally invasive endovascular procedures are transforming the management of PAD. Technologies such as drug-coated balloons and bioresorbable stents promise to improve outcomes for patients with PAD. Drug-coated balloons release medication directly into the affected artery, reducing the risk of restenosis (re-narrowing) after balloon angioplasty. Bioresorbable stents gradually dissolve over time, eliminating the need for permanent metallic implants and potentially reducing long-term complications [67].

Personalized medicine: Personalized medicine is gaining traction in diabetes-related vascular complications. By tailoring treatment plans based on an individual's genetic and molecular profiles, healthcare providers can optimize therapeutic interventions. Personalized approaches can help identify patients more likely to respond to specific medications or interventions, allowing for a more precise and effective treatment strategy. This approach may also lead to fewer adverse effects and better patient outcomes [68].

Bioengineering: Bioengineering can revolutionize the field of limb salvage and replacement. Researchers are working on developing tissue-engineered blood vessels and bioartificial limbs. Tissue-engineered blood vessels aim to provide durable and functional vascular replacements, reducing the need for traditional grafts or amputations. Bioartificial limbs are prosthetic devices that incorporate advanced materials and technologies to mimic natural limb function, offering greater mobility and quality of life for individuals who have undergone amputations [69].

Role of Telemedicine in Diabetes and Vascular Care

Remote monitoring: Telehealth platforms remotely monitor various health parameters, including blood glucose levels, vital signs, and wound progress. Patients with diabetes can use wearable devices and home monitoring equipment to transmit data to their healthcare providers. This real-time monitoring allows healthcare teams to track patients' conditions more closely, detect changes or complications early, and make timely adjustments to treatment plans. For individuals with vascular complications, continuous monitoring of wounds and vascular health can be crucial in preventing complications like infections and amputations [70].

Consultations: Telemedicine consultations offer patients greater access to specialized care, such as vascular assessments and wound consultations. Patients can have virtual visits with vascular surgeons, podiatrists, endocrinologists, and other specialists without needing physical travel. This is particularly beneficial for individuals living in remote or underserved areas where specialized care may not be readily available. Teleconsultations enable timely evaluations and recommendations, helping prevent complications' progression [71].

Education and self-care: Virtual educational programs and telehealth appointments can empower patients to manage their diabetes and vascular conditions better. Patients can receive guidance on medication management, dietary choices, exercise routines, and wound care through telehealth platforms. These interactive programs allow patients to ask questions and receive personalized advice. Educated and engaged patients are more likely to adhere to treatment plans and make healthier lifestyle choices, ultimately improving outcomes [72].

Data integration: Telemedicine platforms often integrate with electronic health records, allowing for seamless coordination between primary care providers, specialists, and wound care teams. This integration ensures that all healthcare professionals involved in a patient's care have access to the same up-to-date information. It reduces the risk of fragmented care and ensures that decisions are made based on current data. Data-sharing among different care team members facilitates collaborative and well-coordinated care [73].

Importance of Multidisciplinary Care Teams

Collaborative approach: Diabetes-related lower extremity vascular complications are complex and multifaceted conditions that require a comprehensive and holistic approach to care. Multidisciplinary care teams bring together healthcare professionals from various specialties, such as vascular surgeons, podiatrists, endocrinologists, nurses, dietitians, and physical therapists. This collaborative approach ensures that all aspects of the condition are addressed. Each team member contributes their unique expertise to develop a well-rounded treatment plan that considers the vascular issues and the patient's overall health, lifestyle, and well-being [74].

Care coordination: Coordination of care is critical in managing diabetes-related vascular complications. Multidisciplinary teams facilitate seamless communication among healthcare providers, reducing the risk of missed interventions or duplicated efforts. For example, a podiatrist may need to work closely with a vascular surgeon to ensure that wound care and vascular procedures are synchronized effectively. Care coordination helps streamline the treatment process, minimizes delays, and optimizes the use of healthcare resources [75].

Patient-centered care: Multidisciplinary teams can tailor treatment plans to meet each patient's needs and preferences. Patients with diabetes-related vascular complications often have unique challenges and circumstances that require personalized care. The care team can consider the patient's medical history, lifestyle, cultural factors, and socioeconomic status by involving specialists. This patient-centered approach increases the likelihood of patient adherence to treatment plans and leads to better overall outcomes [76].

Improved outcomes: Research has consistently shown that multidisciplinary care teams are associated with better clinical outcomes for patients with diabetes-related lower extremity vascular complications. These teams can provide a more comprehensive and integrated approach to care, addressing not only acute issues but also long-term management and prevention. Improved outcomes may include reduced rates of amputation, better wound healing, and enhanced quality of life for patients. Additionally, the efficient use of healthcare resources through coordinated care can lead to cost savings for patients and healthcare systems [77].

Research Gaps and Areas for Future Investigation

Early detection: Developing better screening tools and strategies for the early detection of vascular complications is crucial. Research should focus on non-invasive, cost-effective methods to identify complications at their onset. This could involve advanced imaging techniques, such as ultrasound MRI, or even developing innovative wearable devices that monitor vascular health in real-time [78].

Biomarkers: Identifying biomarkers associated with the development and progression of vascular complications can revolutionize early intervention. Research should aim to discover reliable biomarkers in blood, urine, or other bodily fluids to predict a person's risk of developing complications. These biomarkers could also help tailor treatment plans to individual patients, allowing for more precise and effective care [79].

Patient engagement: Investigating strategies to improve patient engagement and adherence to treatment plans is vital. Behavioral interventions, patient education programs, and digital health technologies can be explored to motivate patients to follow prescribed treatments and lifestyle modifications. Understanding what factors influence patient behavior and how to address them effectively can lead to better long-term outcomes [80].

Health equity: Addressing health disparities in access to care and outcomes among different populations is a pressing research priority. Studies should examine the root causes of these disparities and evaluate interventions to reduce them. This might involve policy changes, community-based healthcare initiatives, and targeted outreach programs to ensure marginalized communities have equal access to preventive care and early intervention services [81].

Long-term follow-up: Long-term follow-up studies are essential to assess the durability and effectiveness of emerging therapies and interventions. Research should track patients over extended periods to determine whether new treatments continue to provide benefits and to identify any potential long-term risks or complications that may arise [82].

Cost-effectiveness: Evaluating the cost-effectiveness of new treatments and technologies is crucial to ensure equitable access to innovative care options. Research should analyze the clinical effectiveness of interventions and their economic impact. Understanding the cost-benefit ratio of different treatments can guide healthcare policy decisions and resource allocation, ultimately benefiting patients and healthcare systems [83].

## Conclusions

Managing diabetes-related vascular complications in the lower extremities is a complex and pressing global health challenge. This comprehensive review has illuminated the multifaceted nature of these complications, from PAD to diabetic foot ulcers and amputations, underscoring their profound impact on individuals and healthcare systems. We have delved into current assessment, diagnosis, and management approaches, emphasizing the crucial role of early detection and comprehensive care. As we navigate the evolving landscape of diabetes care, we must continue prioritizing research and innovation, seeking novel ways to enhance early intervention, promote patient-centered care, and address healthcare disparities. By fostering collaboration across disciplines and embracing emerging technologies, we can strive for better clinical outcomes and improved quality of life for those affected by diabetes-related vascular consequences in the lower extremities. This journey requires dedication, persistence, and a commitment to the well-being of millions, making it a collective responsibility that resonates beyond the confines of this review.
